# [Corrigendum] Inactivation of von Hippel-Lindau increases ovarian cancer cell aggressiveness through the HIF1α/miR-210/VMP1 signaling pathway

**DOI:** 10.3892/ijmm.2026.5983

**Published:** 2026-09-10

**Authors:** Ting Liu, Le Zhao, Wei Chen, Zhen Li, Huilian Hou, Lu Ding, Xu Li

Int J Mol Med 33: 1236-1242, 2014; DOI: 10.3892/ijmm.2014.1661

Following the publication of the above paper, a concerned reader drew the Editor's attention to the fact that a number of data panels shown for the cell migration assay data in Fig. 1C on p. 1238 and Fig. 3A on p. 1240 appeared to contain overlapping sections, such that data which were intended to show the results of differently performed experiments were apparently derived from a much smaller number of original sources.

The authors were contacted by the Editorial Office to offer an explanation for these apparent anomalies in the presentation of the data in this paper, and they have replied to confirm that Figs. 1 and 3 were assembled incorrectly; moreover, although the overlapping images appeared to represent different fields of view, the authors suspected that the errors may have occurred when misfiled images were inserted from the wrong folder during figure preparation. In addition to the issues with Figs. 1 and 3, and even though there were no obvious issues of data mishandling present, the authors also declared that they were unable to retrieve the original experimental data relating to the cell migration assay experiments shown in Fig. 4.

The authors requested that the cell migration experiments pertaining to Figs. 1, 3 and 4 be repeated, and the Editor agreed to the authors' request. The revised versions of Figs. 1, 3 and 4, now showing new data for Figs. 1C, 3A and 4B, together with new histograms that have been prepared for the re-quantification of the new data, are shown on the next two pages. Note that the results obtained from the newly performed experiments were broadly similar to the originally published results, and therefore the revisions made to these three figures have not grossly affected either the results or the conclusions reported in this study. All the authors agree with the publication of this Corrigendum, and they are grateful to the Editor of *International Journal of Molecular Medicine* for granting them the opportunity to publish this Corrigendum; furthermore, they apologize to the readership of the Journal for any inconvenience caused.

## Figures and Tables

**Figure 1:**
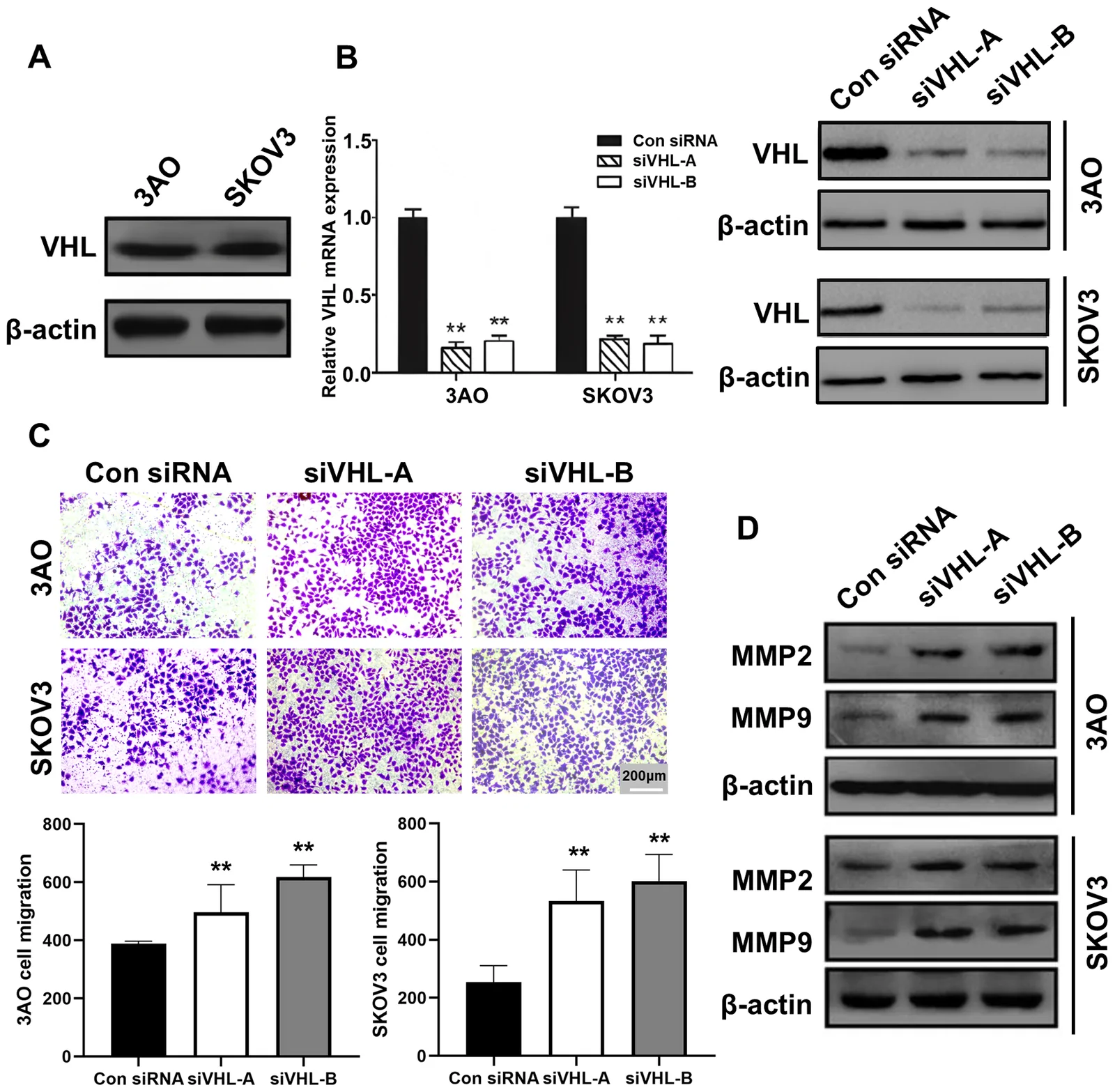
Loss of von Hippel-Lindau (VHL) promotes aggressiveness of ovarian cancer cells. (A) Western blot analysis revealed that the 3AO and SKOV3 cells expressed moderate levels of VHL protein. β-actin was used as an internal control. (B) Detection of interference efficiency of siVHL. Left panel, 3AO and SKOV3 cells were transfected with siVHL for 48 h, and the VHL mRNA level was examined by qRT-PCR. Right panel, cells were transfected with siVHL for 72 h, and the VHL protein level was measured by western blot analysis. A scrambled siRNA was used as a negative control (Con siRNA). (C) Silencing of VHL enhanced the motility of 3AO and SKOV3 cells. Cells were harvested 24 h after siRNA transfection, and placed into Millicell chambers for 12 h to examine the *in vitro* migration ability. (D) Suppression of VHL increased the expression of matrix metalloproteinase (MMP)2 and MMP9. Western blot analysis revealed that the protein level of MMP2 and MMP9 was elevated in the VHL-silenced cells. Data shown are the means ± standard deviation (SD) of 3 independent experiments. ^*^P<0.05, ^**^P<0.01, as shown by Student's t-test.

**Figure 3:**
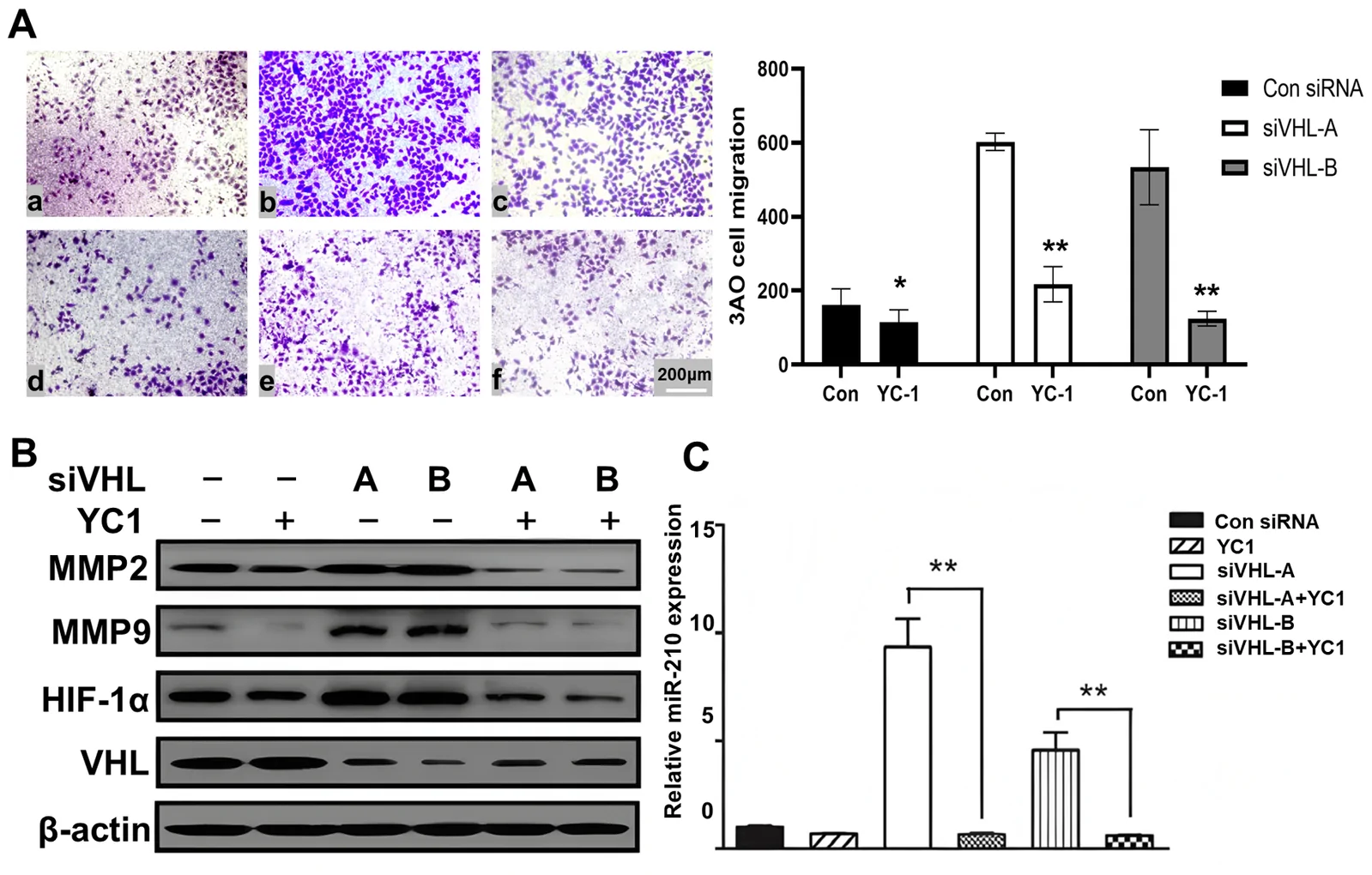
Hypoxia-inducible factor 1-α (HIF-1α) mediates the effects of von Hippel-Lindau (VHL) silencing on cell migration and invasion. (A) *In vitro* migration assay showed that the number of migrated cells significantly increased when VHL was knocked down, which was reversed by the HIF-1α inhibitor, YC-1. (a) negative control (NC); (b) siVHL-A; (c) siVHL-B; (d) YC-1; (e) siVHL-A + YC-1; (f) siVHL-B + YC-1. (B) Western blot analysis revealed that YC-1 inhibited HIF-1α, matrix metalloproteinase (MMP)2 and MMP9 protein expression in VHL-silenced cells. (C) The level of miR-210 was measured by qRT-PCR. The miR-210 expression level was attenuated by YC-1 in the VHL-silenced cells. Values are presented as the means ± standard deviation (SD) from 3 independent experiments. ^*^P<0.05, ^**^P<0.01, as shown by Student's t-test.

**Figure 4:**
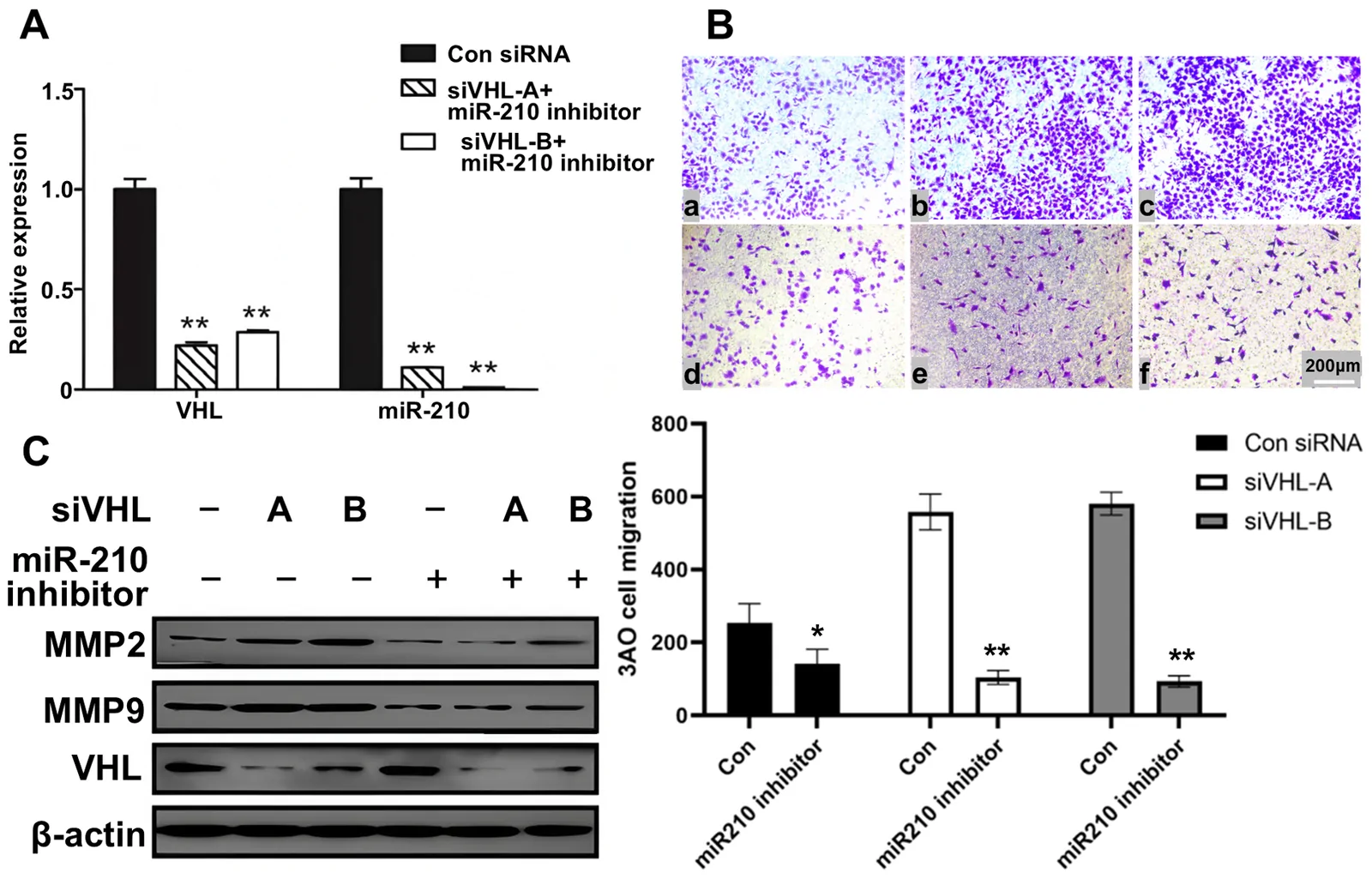
miR-210 is involved in the migration and invasion potential of von Hippel-Lindau (VHL)-silenced cells. (A) The level of VHL mRNA and miR-210 was examined by qRT-PCR in cells co-transfected with siVHL and miR-210 inhibitor simultaneously. (B) *In vitro* migration assay revealed that miR-210 inhibitor repressed the migration ability of VHL-silenced cells. (a) negative control (NC); (b) siVHL-A; (c) siVHL-B; (d) miR-210 inhibitor; (e) siVHL-A + miR-210 inhibitor; (f) siVHL-B + miR-210 inhibitor. (C) miR-210 inhibitor downregulated the protein expression of matrix metalloproteinase (MMP)2 and MMP9. Data are presented as the means ± standard deviation (SD) from 3 independent experiments. ^*^P<0.05, ^**^P<0.01, as shown by Student's t-test.

